# Sleep quality, the neglected outcome variable in clinical studies focusing on locomotor system; a construct validation study

**DOI:** 10.1186/1471-2474-11-224

**Published:** 2010-09-28

**Authors:** Emin Aghayev, Haiko Sprott, Dieter Bohler, Christoph Röder, Urs Müller

**Affiliations:** 1 Institute for Evaluative Research in Medicine, University of Bern, Stauffacherstrasse 78, 3014 Bern, Switzerland; 2 Department Rheumatology and Institute of Physical Medicine, University Hospital of Zürich, Rämistrasse 100, 8091 Zürich, Switzerland

## Abstract

**Background:**

In addition to general health and pain, sleep is highly relevant to judging the well-being of an individual. Of these three important outcome variables, however, sleep is neglected in most outcome studies.

Sleep is a very important resource for recovery from daily stresses and strains, and any alteration of sleep will likely affect mental and physical health, especially during disease. Sleep assessment therefore should be standard in all population-based or clinical studies focusing on the locomotor system. Yet current sleep assessment tools are either too long or too specific for general use.

**Methods:**

Based on a literature review and subsequent patient-based rating of items, an expert panel designed a four-item questionnaire about sleep. Construct validation of the questionnaire in a random sample of the German-speaking Swiss population was performed in 2003. Reliability, correlation, and tests for internal consistency and validity were analyzed.

**Results:**

Overall, 16,634 (70%) out of 23,763 eligible individuals participated in the study. Test-retest reliability coefficients ranged from 0.72 to 0.87, and a Cronbach's alpha of 0.83 indicates good internal consistency. Results show a moderate to good correlation between sleep disturbances and health perception, and between sleep disturbances and overall pain.

**Conclusions:**

The Sleep Standard Evaluation Questionnaire (SEQ-Sleep) is a reliable and short tool with confirmed construct validity for sleep assessment in population-based observational studies. It is easy to administer and therefore suitable for postal surveys of the general population. Criterion validity remains to be determined.

## Background

Just as general health and pain are very relevant outcome variables in judging an individual's well-being, so too is sleep. Yet of these three important variables, sleep is often neglected in outcome studies.

Sleep is a very important resource for recovery from daily stresses and strains, and its alteration can affect both mental and physical health [1-6]. Sleep is even more important during disease. Sleep alterations increase the pathological significance of any disease and reduce general well-being [7-10]. Individuals with altered sleep frequently require pain killers or sleep medication to get decent sleep. Therapeutic sleep restoration is often an important condition for the well-being of patients. Unfortunately, sleep is neglected in most of the frequently used general quality of life (QoL) questionnaires such as the SF36 [11,12], EuroQol 5 D [13], WHODAS II, Health Assessment Questionnaire [14], McMaster Health Index [15], and WHO-5 [16] or assessment instruments focusing on the locomotor system such as the WOMAC [17], Arthritis Impact Measurement Scales [18], Oswestry Disability Index [19], and many others. Among frequently used QoL assessment instruments, the Sickness Impact Profile [20], the WHOQol-100, the Duke Health Profile [21] and the Nottingham Health Profile [22] include one or more questions on sleep characteristics, but they are not meant to be analyzed as an independent dimension of general health.

A number of patient-reported sleep measures have already been published [23]. Measures involving laboratory visits, sleep diaries, or actigraphy consume time and money in clinical trials or population-based studies [24]. Some questionnaires developed for sleep assessment are quite comprehensive [25-28] and too long to be used as population-based questionnaires or in clinical trials where sleep quality is one of many dimensions to be assessed. The Epworth Sleepiness Scale is a short instrument focusing on only one dimension of the sleep problem, daytime sleepiness, which therefore makes it useful only in specific circumstances [29]. The four-item scale of Jenkins et al. [30], although brief, contains no questions about either sleep medication or patient-reported reasons for sleep problems. Such questions are important in patients with disease of the locomotor system because sleep medication is an indicator of the severity of a sleep problem, and patient-reported reasons for sleep problems are needed to discriminate between locomotor, psychological, or other sources of sleep problems.

The need for a very brief, patient-reported sleep measure designed for population-based studies or clinical trials in which sleep is one of several dimensions to be assessed is clear. Such an instrument will encourage inclusion of valid sleep items in clinical epidemiological research. Here we report on the development and construct validation of a Sleep Standard Evaluation Questionnaire (SEQ-Sleep) using a population-based sample. SEQ-Sleep can be combined with the previously published Pain Standard Evaluation Questionnaire (SEQ-Pain) [31] or any other questionnaire.

## Methods

### The Standard Evaluation Questionnaire

The "Standard Evaluation Questionnaire" (SEQ) was developed in 2003 as part of a nationwide, population-based survey in the German-speaking area of Switzerland [31]. SEQ consists of 43 items relating to different ICF (International Classification of Functioning, Disability, and Health) dimensions [32] such as demographic variables, pain, activities of daily living (ADL), medications, need of help, sports, work, smoking and drinking, social conditions, mental health, and sleep.

### Development of the sleep items within the SEQ (SEQ-Sleep)

**Figure 1:**
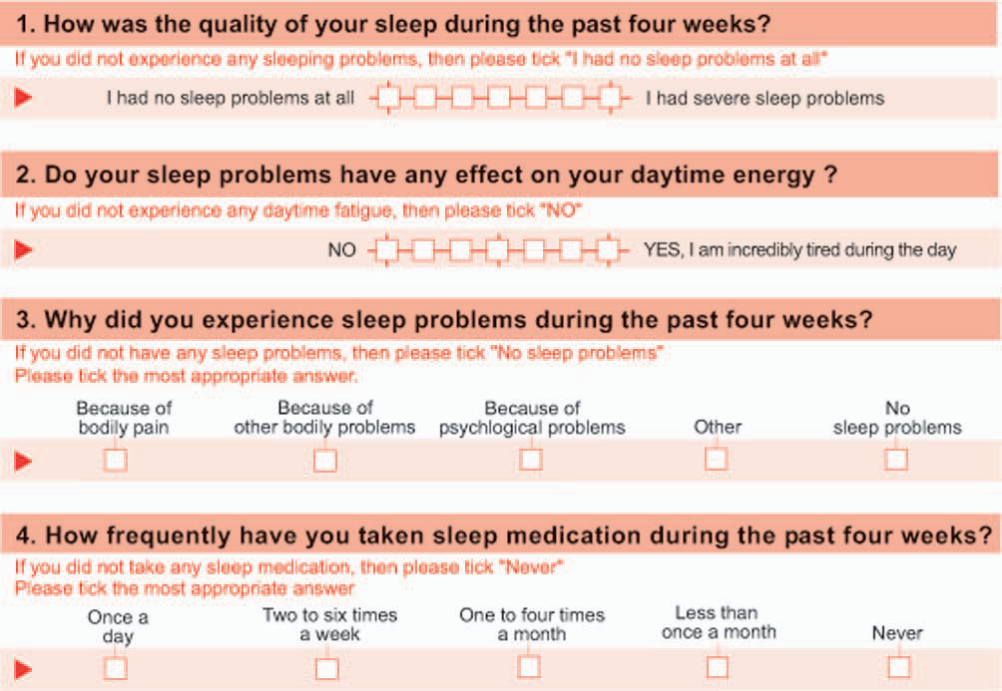
**Final screening tool for evaluation of Sleep (English version not validated yet)**. The answers of the first two mandatory questions are summed to a score (0 = normal sleep, 12 = fully disturbed sleep resulting in severe daytime tiredness). The facultative question 3 asks for the reason sleep is affected, and the facultative question 4 inquires about sleep medication. Questions 3 and 4 are not part of the summed score.

Based on a literature review, two questionnaires were selected for closer scrutiny: the MOS Sleep Scale [28] and the Pittsburg Sleep Quality Index [25]. All items (n = 31) relating to sleep viewed from various perspectives were potentially eligible. Overlapping items were dropped based on expert opinion [33] (12 items). The expert panel included a psychologist, two medical doctors, and a clinical epidemiologist. Retained items were translated into German and rephrased if necessary to achieve a standardized format. The pooled sleep items (n = 19) were then distributed to a set of 20 individuals to rate each question according to subjective importance. For the reduction of the questionnaire the four most important questions (basic questions 1 and 2 selected by the individuals, optional questions 3 and 4 added by the experts) were selected and united with the rest of the SEQ, and pre-evaluation tests were done before moving to a pilot study (Figure 1). Question 4 was closely related to question 6 from the Pittsburg Sleep Quality Index, which was translated to German and validated [34].

### Pilot study

As part of the complete SEQ booklet, the four sleep items were tested in a pilot study on a mixed sample of 637 individuals (out of 1100 individuals approached) from 17 institutions (response rate = 58%). 125 of these participants were enrolled in advanced training courses at three sport schools (response rate within this subgroup = 72%), 171 were employees of four commercial companies (response rate = 67%), 69 lived at homes for the elderly (response rate = 53%), 161 came from a tertiary care emergency department (response rate = 44%), and 111 were patients from six orthopedic or rheumatology tertiary care centers (response rate = 64%). The mean age of participants (53% women) was 47.2 ± 20 and mean body mass index was 23.8 kg/m^2 ^± 4. Musculoskeletal complaints were reported by 56%, and 9% were dependent on help for daily living. The first 48 responders were asked to take part in a structured interview aimed at identifying ambiguities in wording and adapting the relevant items (45, or 94%, participated).

A computer generated random sample of 250 participants was included in a test-retest study (n = 249; 99%). The time lag between the two questionnaires was seven days on average (range 4-11). The intraclass correlation coefficient (ICC, calculated using bootstrap method for two-way mixed-effects ANOVA ICC) for the two items in the sleep sections was 0.84, and weighted kappa coefficients ranged from 0.71 to 0.79 (median 0.78) and were independent of age, gender, and section.

### Participants and mailing procedures during the main construct validation study

**Figure 2:**
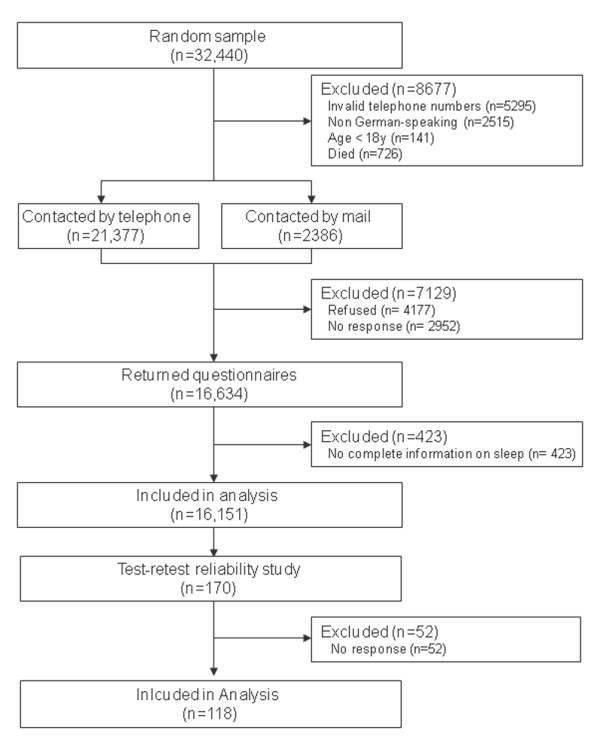
**Study flowchart**.

A randomly generated total of 32,440 private households in the German-speaking part of the Switzerland were selected from the telephone directory. 8,677 households were excluded: 5,295 had invalid phone numbers, 2,515 were unable to understand German, 726 individuals living alone were reported as deceased, and 141 individuals were below 18 years of age (Figure 2). A total of 23,763 households were therefore eligible for a telephone interview and 21,377 (90%) of these were contacted between November 2002 and June 2003. Potential participants were informed about the study and consent to receive a postal questionnaire was obtained. In multiple-member households, the last-birthday selection technique was used in analogy to the next-birthday method to identify one participant per household aged 18 years or older who was able to understand German [35]. Overall, 17,341 of the 21,377 individuals (81%) consented to participate during the telephone interview. A cover letter with additional study information, a questionnaire, and a prepaid return envelope were then sent to them. About 10% (2386) of the 23,763 households could not be reached by phone after a maximum of 12 attempts and were contacted by mail using an extended cover letter. Nonresponders were sent a reminder letter after three weeks, or after six weeks a reminder letter, the questionnaire, and a prepaid return envelope were delivered by priority mail.

### Testing and validation

The stepwise approach described by Streiner & Norman [36] and Veenhof et al. [37] was used to select and evaluate items. More precisely, item selection for the definitive questionnaire was a three-step procedure relying upon 1) test-retest reliability (intra-item reliability), 2) inter-item correlations or redundancy, and 3) Pearson product-moment correlation between sleep items, pain intensity (relevancy), and general health. The first question of the SF-12 questionnaire was used as the general health measure ("In general, would you say your health is?"). All steps were performed in all samples, with a subsequent sensitivity analysis restricted to individuals reporting pain in any location on the numeric rating scales in the original Standard Evaluation Questionnaire.

### Test-retest reliability

The test-retest reliability was assessed by sending a second questionnaire to the first 170 responders within a scheduled interval of 7 to 14 days as described by Müller et al. [31]; ICCs and weighted kappa statistics were used. Values larger than 0.60 were considered to be substantial [38] and items were discarded if the estimated ICCs or kappa values were below 0.60 [39].

### Inter-item correlation

Potential redundancy between items extracted from the same domain was identified by assessing the correlation between items. If Pearson product-moment correlation between any two items was larger than 0.75, one of the items was discarded.

### Internal consistency

Cronbach's alpha was determined for the scale as a measure of overall consistency of the two items, and the item total correlation between each item and the total score was calculated.

### Association with intensity of pain and general health perception (relevancy)

We aimed to show the relevancy of musculoskeletal pain problems to sleep. Measurement of overall pain in different body regions was conducted by using the same distinction described by Müller et al. [31] and the maximum reported value was used. For example, if an individual reported a pain rating of six in the right leg and three in the back, a rating of 6 was assigned. The Pearson product-moment correlation coefficient was used to determine correlations between ratings of the higher pain reported in body regions (head and face, upper extremity, neck and back, chest and stomach, lower extremity) and the ratings of other, remaining items to determine the relevancy of the items for pain. Items were discarded if the correlation coefficient was below 0.25. Additionally, some descriptive statistics will be reported to support the relevancy of measuring sleep perception.

The relationship between musculoskeletal pain and sleep problems was analyzed. Comparison of patients with pure musculoskeletal pain with and without sleep problems was performed using a Chi-square test.

For assessment of the sincerity and accuracy of the participants in answering question 3, their answers were compared to reported pain intensity.

### General issues

Responses were digitized by an independent institution using optical character recognition and all analyses were performed in Stata 10.0.

## Results

### Characteristics of participants

Of the 23,763 individuals eligible for a telephone interview, 16,634 (70%) responded while 4,036 (17%) declined participation during the phone interview; 3,093 (13%) failed to return the questionnaire. No information on pain was provided in 443 questionnaires that therefore were excluded from the analysis. The exclusion of those patients did not significantly change the distributions of patients among the various groups of different patient characteristics. 264 of the 16,191 individuals (1.6%) did not fully complete the SEQ-Sleep (Figure 2). Participant characteristics are presented in table 1. Mean age was 49.1 ± 16.9 years, the mean body mass index was 24.5 ± 4.2, and 58% were women.

**Table 1:** Characteristics of participants.

	Participants included in analyses (n = 16'191)	Participants reporting sleep problems (n = 8297)	Participants reporting pain (n = 10'610)
Females (n[%])	9256 (58%)	4923 (59%)	6316 (60%)
Age band (n[%])			
18 to 34 years	3554 (23%)	1812 (22%)	2194 (21%)
35 to 44 years	3691 (23%)	1937 (23%)	2393 (23%)
45 to 54 years	2797 (18%)	1488 (18%)	1901 (18%)
55 to 64 years	2522 (16%)	1369 (16%)	1782 (17%)
65 to 74 years	1957 (12%)	969 (12%)	1306 (13%)
75 to 84 years	1136 (7%)	678 (8%)	718 (7%)
85 years and above	220 (1%)	94 (1%)	127 (1%)
Body mass index			
Less than 25 kg/m^2^	9728 (62%)	5045 (62%)	6239 (60%)
25 to 30 kg/m^2^	4633 (29%)	2323 (29%)	3098 (30%)
30 kg/m^2 ^and above	1441 (9%)	771 (9%)	1036 (10%)
Highest educational level (n[%])			
Compulsory schooling/vocational training	9760 (61%)	4843 (59%)	6364 (60%)
High school graduation	2141 (13%)	1181 (14%)	1476 (14%)
Technical college/university degree	4290 (26%)	2273 (27%)	2850 (27%)
Living independently (n[%])	15'140 (93%)	7641 (92%)	9876 (92%)
Rural residence* (n[%])	11'694 (74%)	6089 (73%)	7765 (75%)
Reported sleep problems (n[%])	8297(51%)	8297(100%)	6247 (59%)
Reported reason for sleep problems (n[%])			
Because of bodily pain	1271 (8%)	1271 (15%)	1127 (11%)
Because of other bodily problems	407 (3%)	407 (5%)	340 (3%)
Because of psychological problems	1572 (10%)	1572 (19%)	1259 (12%)
Because of other problems	5047 (30%)	5047 (61%)	3521 (33%)
Reported complaint or condition (n[%])			
Musculoskeletal	9603 (59%)	5529 (67%)	8583 (80%)
Visual or hearing	5946 (37%)	3340 (40%)	4296 (40%)
Cardiovascular	2771 (17%)	1480 (18%)	1946 (18%)
Nervous system	4346 (27%)	2737 (33%)	3640 (34%)
Respiratory system and allergies	4051 (25%)	2281 (27%)	2877 (27%)
Mental	1434 (9%)	1078 (13%)	1151 (11%)
Gastrointestinal	1886 (12%)	1276 (15%)	1620 (15%)
Diabetes	472 (3%)	241 (3%)	319 (3%)
Renal	390 (2%)	237 (3%)	291 (3%)
Neoplasia	446 (3%)	256 (3%)	328 (3%)
Currently smoking (n[%])	4078 (26%)	2086 (25%)	2702 (26%)
No sleeping problems	8197 (50%)	0 (0%)	4354 (41%)

* Village of less than 10'000 inhabitants

### Test-retest reliability

The second test-retest study questionnaire was by returned by 118 (69%) of the first 170 responders within 5 to 13 days (median 11); 77 (65%) of these were women with a mean age of 52.2 ± 16.0 years. The ICC between the original and retest ratings for item one was 0.87 and 0.85 for item two. The median kappa value between the original and the retest item was 0.78 for question three and 0.72 for question four, exceeding the lower limit for inclusion of 0.60.

For the subsample of patients with sleep problems due to musculoskeletal pain, the ICC between the original and retest ratings for item one was 0.83 and 0.84 for item two. The median weighted kappa value between the original and the retest item was 0.77 for question three and 0.74 for question four.

### Inter-item correlation: Redundancy

Inter-item correlations of questions 3 and 4 were moderate both for the overall sample (0.71) and those reporting pain (0.70). All values were below the upper bound for inclusion of 0.75.

### Internal consistency

We analyzed internal consistency for variables one and two (sleep quality and the effects of sleep problems on fatigue) using Cronbach's alpha as well as the item total correlation between each item and the sleep score (Table 2). The values can be considered to be high, especially since only two items were used.

**Table 2:** Internal consistency regarding to the overall sample and the subsample reporting bodily pain (Internal consistency: Cronbach's alpha of the first two items building the total score).

Consistency: Modalities and characteristics of the sleep items	All participants *(n = 16'191)*		*Participants* *reporting* *pain* *(n = 10'610)*		*Participants with sleep* *problems only because* *of bodily pain* *(n = 899)*	
	ICC	Cronbach's α	ICC	Cronbach's α	ICC	Cronbach's α
*Sleep*		*0.83*		*0.82*		*0.79*
Sleep quality	0.89		0.89		0.85	
Effects of sleep problems on fatigue	0.86		0.85		0.83	

### Association with intensity of pain and general health perception: relevancy

**Figure 3:**
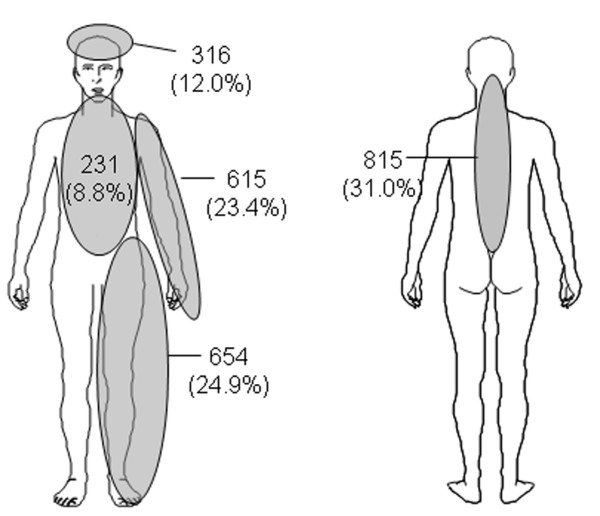
**Frequency of pain locations (n = 2631) in the 1271 individuals that suffered from sleep reduction because of bodily pain**.

Pearson product-moment correlation coefficients between quantitative items of sleep with the overall intensity of pain and general health were moderate (Table 3). The correlation between sleep problems and pain intensity increased to 0.71 if calculated for the subcollective indicating bodily pain as the primary reason for disturbed sleep (n = 899). Back pain was the main source of sleep disturbance in this sample (Figure 3).

**Table 3:** Relevance: How does the sleep score correlate with overall pain and general health?

Relevancy: Pearson product-moment correlation coefficients	All participants *(n = 16'191)*		*Participants* *reporting pain* *(n = 10'610)*		*Participants with sleep* *problems only because* *of bodily pain* *(n = 899)*	
	Overall pain	General health	Overall pain	General health	Overall pain	General health
*Sleep*	*0.34*	*0.33*	*0.30*	*0.32*	*0.71*	*0.4*
Sleep quality	0.33	0.34	0.29	0.33	0.69	0.36
Effects of sleep problems on fatigue	0.30	0.27	0.27	0.26	0.68	0.32

The relationship between sleep problems and musculoskeletal pain is shown in table 4. The table shows a significantly higher proportion of patients with pure musculoskeletal pain with sleep problems because of pain (95%) in comparison with the patients with sleep problems due to reasons other than pain (86%), or with the patients without sleep problems (88%) (chi-square test: p > 0.001). The highest pain intensity of 3.5 points was seen in patients with sleep problems because of pain and the lowest (5.7 points) in patients without sleep problems (Table 4).

**Table 4:** Relationship between sleep problems and musculoskeletal pain.

	N of patients with pain (%)	N of patients without pain (%)	Proportion of patients with pure musculoskeletal pain	Pain intensity (scale: between 1-strong and 7-none)
- No sleep problems	4786 (59%)	3396 (41%)	88%	5.7
- Sleep problems not because of pain	4900 (77%)	1429 (23%)	86%	4.9
- Sleep problems because of pain	1224 (96%)	47 (4%)	95%	3.5
- Sleep problems because of pain (ex. confounding causes*)	899 (96%)	32 (4%)	95%	3.6

In patients with sleep problems because of pain a significantly higher proportion of patients with pure musculoskeletal pain (95%) in comparison with the patients with sleep problems due to reasons other than pain (86%) or with the patients without sleep problems (88%) was seen (p > 0.001). Similar results show patients with sleep problems because of pain without confounding causes (* - patients with the reason for sleep loss other than pain are excluded).

## Discussion

### Summary

The brief Sleep Standard Evaluation Questionnaire, which includes four items to assess sleep in population-based observational studies, was developed and validated. The questionnaire accounts for sleep and its influence on daytime activity (mandatory questions), reasons for sleep problems and frequency of sleep medication (facultative questions). These were shown to be reliable and valid in a large, representative sample of the general Swiss population.

### Strengths and weaknesses

Items selected for inclusion in the questionnaire were extracted from scales previously developed for sleep assessment in clinical trials [25,28]. We did not include items from sleep-assessment scales used in other medical specialties (e.g. neurology or oncology), nor did we develop any new items. According to the guidelines for assessing the quality of scales, a set of patients was involved in the selection of items to be included in the questionnaire [37,39]. A pool of items was proposed by a group of experts from a literature review based on two frequently used sleep questionnaires. Patients' pre-evaluation of the subjectively important sleep items allowed for subsequent reduction of the number of items to a minimum set of four questions. The subsequent study drew a large number of participants from the general population and was not restricted to individuals experiencing disturbed sleep. The validation study also included a sizeable subsample used for calculating test-retest reliability 15% of the participants reporting sleep problems cited bodily pain as the reason for their sleep problems, while 11% of those reporting pain cited pain as the origin of sleep disturbance (Table 1). These figures draw upon a population-based sample in which the majority of persons report only low-level pain and patients with severe pain are rare [31]. It is expected that patients with severe pain will report a higher percentage of sleep disturbances due to bodily pain.

The median duration of 11 days between the completion of the first and second questionnaires in the test-retest study may be too long for assessing the reliability of sleep assessments since acute sleep disturbance episodes could have occurred or sleep quality may have changed during this period. However, test-retest reliability was high for all items. Our questionnaire covers participant experience during the previous four weeks. Acute episodes of increased sleep disturbances and variations in sleep medications might therefore have been unlikely to be detected.

Concurrent validity criterion with respect to other sleep questionnaires was not assessed in our study set up. Our validation study has therefore preliminary character.

As in other surveys, women and individuals with higher education were overrepresented in our study [40]. Other demographic characteristics differed only slightly between our sample and the general Swiss population. The overall response rate in this sample of the general population was 70%, which is higher than other studies [41,42]. The proportion of fully completed questionnaires also was high, indicating that participants carefully responded to the questions. Both of these facts confirm the quality of the data and the suitability of our questionnaire for population-based observational studies. Although the development of the SEQ-Sleep was based on a large population-based sample, it is from only one country with one language. Its transferability to other countries and languages remains unclear.

### Context

Other sleep assessment tools cited above were developed specifically for clinical trials and are not particularly recommended for population-based observational studies [23]. The MOS Sleep Scale, for example, may be used for sleep assessment in restless leg syndrome [43], diabetic peripheral neuropathy [44], neuropathic pain [28], or cancer pain [28,45] where specific sleep assessment is required. Some questionnaires, e.g., the MOS Sleep scale, can be definitively applied for an unspecific sleep assessment. But to our knowledge there is no other short and easy to administer sleep assessment instrument available for the assessment of sleep regardless of the underlying pathology that can provide patient-reported reasons for the sleep problems (e.g., locomotor system pain).

The relationship between general health perception and sleep is expectedly high [46]. Empirically, the restoration of healthy sleep conditions in patients has a high impact on their general health and quality of life perception. This relationship is supported by our results, but needs further investigation.

The patients with sleep problems because of pain have significantly more pure musculoskeletal pain in comparison with the other patients. This underlines important relationship between disturbed sleep and musculoskeletal pain and reasons the use of sleep as an outcome variable in clinical studies on the locomotor system.

### Implications for future studies

For the concluding validation, concurrent validity criterion needs to be determined. Our questionnaire was developed for population-based cross-sectional studies of sleep assessment, but it also could be used in clinical trials and other longitudinal studies. This questionnaire might also be useful in evaluating patients in routine clinical practice and in computer-assisted telephone surveys. Additional study would be required to evaluate the usefulness and psychometric properties of the questionnaire in these settings, even if they might differ only slightly. Our study being restricted to Switzerland and its German-speaking population, the transferability of the questionnaire to other countries and languages needs to be examined. While a validation of the French version is currently underway, future studies also should include validations of English and Spanish translations. Finally, the association between measures derived from our questionnaire and physical or mental conditions merits further study.

## Conclusions

The Sleep Standard Evaluation Questionnaire is a reliable and short tool with confirmed construct validity for sleep assessment in population-based observational studies. It is easy to administer and therefore suitable for postal surveys of the general population. The performed validation has preliminary character as the concurrent validity criterion with respect to other sleep questionnaires is not yet determined.

## Abbreviations

SEQ: Standard Evaluation Questionnaire; QoL: Quality of Life; SF36: a questionnaire on health-related quality of life; EuroQol 5 D: European Quality of Life questionnaire; WHODAS II: World Health Organization Disability Assessment Schedule II; WHO-5: Well Being Index; WOMAC: standardized questionnaire to evaluate the condition of osteoarthritis patients developed at Western Ontario and McMaster Universities; WHOQol: World Health Organization Quality of Life; ADL: Activities of Daily Living; MOS sleep scale: sleep scale from the Medical Outcomes Study; ICC: Intraclass Correlation; NRP: National Research Program

## Competing interests

The authors declare that they have no competing interests.

## Authors' contributions

EA participated in conception of the study, drafting the manuscript, the literature review and the statistical analysis. HS and DB conceived the study and participated in the statistical analysis, data collection, and drafting the manuscript. CR supervised the study and drafting the manuscript. UM conceived the study and participated in coordination and supervision of the study. All authors participated in the study design, and reading and approval of the final manuscript.

## Pre-publication history

The pre-publication history for this paper can be accessed here:

http://www.biomedcentral.com/1471-2474/11/224/prepub
